# Mechanical and Biocompatibility Properties of 3D-Printed Dental Resin Reinforced with Glass Silica and Zirconia Nanoparticles: In Vitro Study

**DOI:** 10.3390/polym15112523

**Published:** 2023-05-30

**Authors:** Abdullah Alshamrani, Abdulaziz Alhotan, Elizabeth Kelly, Ayman Ellakwa

**Affiliations:** 1 Oral Rehabilitation & Dental Biomaterial and Bioengineering, The University of Sydney, Sydney 2006, Australia; 2 Department of Dental Health, College of Applied Medical Sciences, King Saud University, Riyadh P.O. Box 12372, Saudi Arabia; 3 The Cellular and Molecular Pathology Research Unit, Oral Pathology and Oral Medicine, School of Dentistry, The University of Sydney, Westmead Hospital, Westmead 2145, Australia

**Keywords:** additive manufacturing, three-dimensional printing, temporary crown, mechanical properties

## Abstract

This study aimed to assess the mechanical and biocompatibility properties of dental resin reinforced with different nanoparticle additives. Temporary crown specimens were 3D-printed and grouped based on nanoparticle type and amount, including zirconia and glass silica. Flexural strength testing evaluated the material’s ability to withstand mechanical stress using a three-point bending test. Biocompatibility was tested using MTT and dead/live cell assays to assess effects on cell viability and tissue integration. Fractured specimens were analysed using scanning electron microscopy (SEM) and energy-dispersive X-ray spectroscopy (EDS) for fracture surface examination and elemental composition determination. Results show that adding 5% glass fillers and 10–20% zirconia nanoparticles significantly improves the flexural strength and biocompatibility of the resin material. Specifically, the addition of 10%, 20% zirconia, and 5% glass silica by weight significantly increases the flexural strength of the 3D-printed resins. Biocompatibility testing reveals cell viabilities greater than 80% in all tested groups. Reinforced 3D-printed resin holds clinical potential for restorative dentistry, as zirconia and glass fillers have been shown to enhance mechanical and biocompatibility properties of dental resin, making it a promising option for dental restorations. The findings of this study may contribute to the development of more effective and durable dental materials.

## 1. Introduction

In the past few years, there has been rapid advancement in the field of three-dimensional (3D) printing. This technology has become more precise and dependable, making it a desirable choice for dental and medical applications. Three-dimensional printing has been utilised for new applications in many healthcare sectors, including medicine, dentistry, orthopaedics, and medical devices [1,2,3]. The process of transforming digital 3D models into tangible objects can be quickly accomplished using this technology. First, a digital file is created in standard tessellation language (STL) format, and then the design is printed through the joining, bonding, or polymerization of small-volume elements [4]. Many types of 3D printing techniques have been developed and utilised for different dental purposes, including, but not limited to, stereo-lithography (SLA), digital light projection (DLP), fused deposition modeling (FDM), powder bed fusion (PBF), and inkjet printing [5,6].

Choosing the appropriate 3D-printing material for dental purposes depends on the intended use of the final product. For example, dental restorations require materials with strong mechanical properties and slow biodegradation rates to withstand the forces generated during chewing. Additionally, successful dental restorations rely on materials that can integrate well with the oral tissue [7]. When designing dental restorations, especially temporary crowns, it is crucial to consider all the significant requirements, such as superior mechanical and physical properties, appropriate biocompatibility, ease of handling, and cost-effectiveness of the material. Polymer-based materials are popular resins that are used to produce dental prostheses using additive technology. Composite resin-based materials are frequently used to fabricate provisional restorations. These materials consist of filler particles, a coupling agent, a resin matrix, and a catalyst, all of which are blended to create a uniform mixture [8].

Consequently, to enhance the performance of provisional dental resins, which include improvement of longevity and biocompatibility, various materials have been used to reinforce dental-based composites, such as metals, fibres, and various oxides (aluminum, zirconium, and titanium), which have resulted in both positive and negative outcomes [9,10,11,12,13,14,15]. Most of these recent attempts focused on modifying the resin matrix by incorporating nanofillers and particles to create nanocomposites with better properties [16]. One way to increase the filler content and, hence, the mechanical properties is the addition of micro-fillers [17] or pre-polymerised resin fillers into the resin matrix of microfilled composites [18]. Many studies show that modifying dental resins with various fillers (usually micro-sized and nano-sized) significantly improves their mechanical, physical, and biological properties [14,15,19,20]. However, some adverse effects have been reported, such as the creation of voids and porosity, reduced biocompatibility, and a degree of polymerisation over time [21,22,23].

A group of researchers in dentistry studied how adding zirconium oxide (ZrO_2_) particles could enhance the mechanical properties of resin-based materials [24]. They examined parameters such as surface treatment, particle size, and the dispersive method. The findings show that adding ZrO_2_ particles improves properties such as the hardness and flexural strength [23]. In addition, the distribution of particles also affects the flexural strength. Another study found that a combination of ZrO_2_ nanoparticles and glass fillers significantly improved the flexural and impact strengths of dental resins [22]. The addition of glass silica and zirconia nanoparticles to the dental resin has been found to significantly enhance its hardness and wear resistance. Glass silica nanoparticles im-prove the material’s hardness by creating a more compact and dense structure, increasing its resistance against deformation and wear [25,26]. In contrast, zirconia nanoparticles reinforce the resin matrix, further enhancing its hardness and wear resistance [27]. Reinforcement reduces the risk of wear, chipping, or fractures over time, contributing to the longevity of dental restorations. These combined effects make the incorporation of these nanoparticles a promising approach for improving the mechanical properties of dental resins [26,28].

The inclusion of fillers in printable resins can increase the viscosity, leading to poor printability and issues such as clogging, uneven flow, and decreased accuracy [29,30]. The viscosity of the uncured resin must be as low as is feasibly possible to enable adequate recoating of the liquid monomer on the polymerised underlayer between the vat’s surface, and the building platform fillers can also settle over time, causing uneven distribution in the resin and inconsistent mechanical properties in printed objects [31]. Careful consideration of the filler type, size, and concentration is crucial to avoid these challenges and achieve optimal printability and material properties in printable resins.

Dental resins are commonly used in different applications, such as denture base materials, liners, and dental crowns. However, increasing concerns have arisen regarding the biocompatibility of these resins and their safety for clinical application, owing to the release of potentially toxic compounds from the polymer network and changes in the physical and mechanical properties of the material under oral conditions [32]. The interim restoration resin material may contain high levels of unpolymerised monomers, which could harm the restoration’s physical properties and biocompatibility [33]. These residual and leachable monomers have the potential to enter the oral cavity and cause cytotoxicity in the tooth pulp and to surround the gingival soft tissue that comes into contact with the 3D-printed restoration [34,35].

It is also crucial to carefully consider the biocompatibility of any material used in the mouth, including temporary restorative resins. It is essential for materials used in the mouth to be biocompatible in order to minimize the risk of adverse reactions and not adversely affect cell growth, tissue integration, or overall patient safety. This requires the use of resins with appropriate material properties, such as biodegradability, non-toxicity, and suitable mechanical, chemical, and biological properties, to meet the specific needs of biomedical applications.

Post-processing is critical for biocompatibility [36,37]. Additional curing, cleaning, and surface treatments are typical post-processing procedures for 3D-printed resins [38]. Cleaning is essential for thoroughly removing any residual uncured resin, solvents, or support structures to ensure the purity of printed objects [39]. Moreover, surface treatments, such as polishing, sanding, and coating, play a crucial role in achieving the desired surface quality and functionality of printed objects while maintaining their biocompatibility for biomedical applications [40]. These post-processing processes are critical for improving the overall performance and usability of 3D-printed resins for various biomedical applications.

The lack of studies on the biocompatibility of plasticisers and residual monomers in temporary restorative resin materials can be attributed to the absence of comprehensive information provided by manufacturers regarding the composition of these materials. This gap in knowledge regarding these components and their impact on biocompatibility has resulted in a limited understanding of the potential risks associated with the use of such materials in dental practice. This lack of information makes it difficult for researchers to fully understand their biocompatibility. Therefore, manufacturers must provide detailed information about the components of their products to facilitate further research on the biocompatibility of these materials.

Further investigation is needed to explore the manufacturing process and impact of nanoparticle reinforcement on the mechanical and biological characteristics of 3D-printed dental materials. The current study evaluated the effects of adding glass fillers and zirconia nanoparticles to dental resins on the flexural strength and biocompatibility of DLP-printed temporary crown resin materials. The null hypothesis was that the flexural strength and biocompatibility properties of the 3D-printed resin would not significantly affect the incorporation of the reinforced glass silica.

## 2. Materials and Methods

### 2.1. Sample Preparation and 3D-Printing

The 3D-printed reinforced composites were obtained by incorporating silane-coated glass fillers (ultrafine GM35429) with an average particle size of approximately 1.5 µm and silane-coated zirconia particles (ultrafine G018307) with an average particle size of approximately 0.4 µm (Schott, Landshut, Germany) at concentrations of 5%, 10%, and 20% (*w*/*w*). To ensure the dispersion performance of glass silica and zirconia nanoparticles in dental resin, the nanoparticles were added to the printable resin solutions and stirred for 24 h using a magnetic stirrer. The mixture was then sonicated for 45 min in a water bath and dispersed in a 3D-printed resin to help break up the agglomerates and promote uniform dispersion. Table 1 presents a comprehensive list of the materials used in this study.

**Figure 1:**
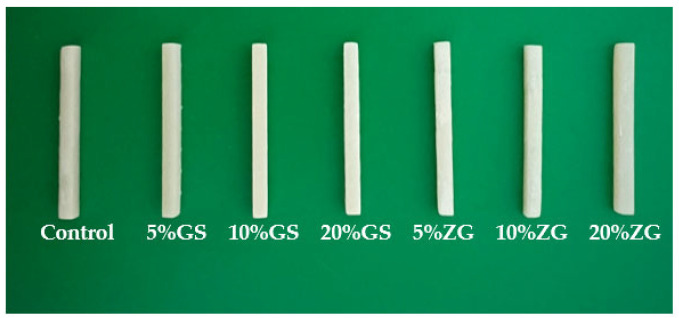
Representative 3D-printed samples of control (unmodified 3D-printed resin), glass silica 5 wt% (GS 5%), glass silica 10 wt% (GS 10%), glass silica 20 wt% (GS 20%), zirconia 5 wt% (Zir 5%), zirconia 10 wt% (Zir 10%), and zirconia 20 wt% (Zir 20%).

The printing process for bar-shaped specimens of light-cured resin material (B2 Everes Temporary, Sisma, Italy) used for provisional restorations is as follows. Firstly, the digital data of the bar-shaped sample in STL format were exported and imported into Asiga Composer Software (Asiga HQ, Alexandria, NSW, Australia). The dimension used was (25 × 2 × 2 mm) in line with ISO 4049 (International Organization for Standardization, 2019). The printing settings were then configured, including a print orientation of 90 degrees from the print area, based on accuracy evaluations of thickness, width, and length from a previous study [38]. Subsequently, automated printing supports were generated at the bottom of the bar-shaped sample, with a point size of 0.5 mm, density of 0.85, and height of 3.0 mm. These settings were replicated multiple times to create the necessary number of samples for testing. The samples were then placed on the build platform of the Asiga MAX DLP printer (Asiga, Alexandria, NSW, Australia) to print using the same configuration. The printer employs an LED light source operating at a wavelength of 385 nm. After printing, the specimens were washed in 90% isopropyl alcohol for 5 min, following the manufacturer’s instructions. A scraper was used to delicately remove the specimens off the construction platform. A second rinse with fresh isopropanol was used to entirely eliminate any leftover uncured monomers on the surface. The specimens were dried with compressed air. The post-curing procedure was conducted utilizing a light curing unit with a broad wavelength spectrum of 400–550 nm (Solidilite V, Shofu Dental GmbH, Ratingen, Germany) for a duration of 10 min to facilitate the polymerization process. The sample size for the study was determined based on the results of a previous study, resulting in a minimum sample size of 10 per group [41]. Each group was printed at each percentage to have a total of 105 specimens for the study, with 70 samples for flexural strength (*n* = 10) and 35 samples for cell viability (*n* = 5). The final printed samples have been visually captured and are presented in Figure 1.

### 2.2. Flexural Strength Test

To conduct the three-point bending test, a universal testing machine (compliant with ISO standard 4049) was utilised. The specimens were secured between two supports with a 20 mm span and loaded at a crosshead speed of 0.5 mm/min until they fractured. The flexural strength was determined by taking into account the load at which the fracture occurred and the dimensions of the specimens, which were measured using a digital calliper. The flexural strength (σ) was then calculated in megapascals using the following formula [42]:

σ = 3 FL/2 wd^2^ (1)

where σ is flexural strength, F is load at the fracture point, L is length of the support span, w is the width of specimen, d is the thickness of the specimen, and d is the deflection of the specimen in millimetre.

### 2.3. Biocompatibility Test

#### 2.3.1. In Vitro Cell Culture

A gingival fibroblast cell line was used in this study (PCS-201-018 IS ATCC). The experimental procedures involved culturing fibroblasts in DMEM (Thermo Fisher Scientific Waltham, MA, USA) supplemented with 10% fetal bovine serum (FBS; Thermo Fisher Scientific Waltham, MA, USA), as well as antibiotics penicillin (60 µg/mL), streptomycin (100 ug/µL), and L-glutamine (2 mM) in a humidified atmosphere maintained at 37 °C and 5% CO_2_. Only cells from the second to fourth passages were used for the experiments.

#### 2.3.2. Eluent Preparation

The preparation of extracts from each group of samples was performed following international standards (Iso, 2009). A total of 21 samples of 3D-printed composite resin were divided into a control group of unmodified resin material (*n* = 3) and six groups (*n* = 3) of modified 3D-printed resin with either glass silica or zirconia fillers of 5, 10, and 20 wt%. A culture medium containing 10% FBS was placed in a sterilised glass bottle, and the appropriate amount of 3D-printed resin was added to it. The extraction ratio followed the ISO 10993-12 guidelines and was set to 0.2 g/mL. The samples were then incubated at 37 °C and 5% CO_2_ in a humidified environment for 24 h. Subsequently, the culture medium containing the material extracts was filtered using a 0.22 µm cellulose acetate filter (Mil-lipore, Sigma, St. Louis, MO, USA), and the extracted solution was utilised for the MTT assay.

#### 2.3.3. MTT Assay

The MTT assay is a colorimetric assay used to measure cell viability. This test was conducted in accordance with ISO 10993-5. The gingival fibroblast cells were seeded into 24 well plates (Costar, Kennebunk, ME, USA) at a density of 1 × 10^4^ cells/well and incubated in culture for 24 h to form a semi-confluent monolayer. After that, the media was removed from the wells and replaced with conditioned media (sample extraction) at a volume of 500 µL volume in each well.

Following a 24 h incubation period, the culture medium was replaced with 50 µL of MTT solution (1 mg/mL in phosphate-buffered saline or PBS). Subsequently, the MTT solution was discarded, and 100 µL of isopropanol was added to each well. To determine the endpoint of the MTT assay, the absorbance of each well was measured at 570 nm using a microplate reader (Epoch, BioTek, Winooski, VT, USA). Each group was tested in triplicate for the assay. The mean survival rate after 24 h was compared to the positive control group with cell culture medium, and the viability response to the 3D-printed resins was rated as non-cytotoxic (>85% survival), slight (60–85% survival), moderate (30–60%), or severe (>30% survival) [43]. The percentages viability was calculated as follows:

(2) $$\mathrm{Cell}\;\mathrm{viabilty}\;\left(\%\right)=\frac{{\mathrm{OD}}_{\mathrm{test}\;\mathrm{sample}}-{\mathrm{OD}}_{\mathrm{blank}}}{{\mathrm{OD}}_{\mathrm{PC}}-{\mathrm{OD}}_{\mathrm{blank}}\;}\times 100$$

In the provided equation, the variable “OD” represents the measured optical density of each sample. “OD_test sample” refers to the optical density value obtained from the sample being tested. “OD_blank” corresponds to the background optical density, typically acquired from a blank sample that does not contain cells. “OD_PC” represents the optical density of a positive control that is cells in DMEM with 10% FBS that serves as a reference of 100% cells viability.

#### 2.3.4. Live and Dead Assay

The viability of cells was determined using a live/dead staining assay (Invitrogen^TM^, Waltham, MA, USA) according to the manufacturer’s protocol. Calcine AM (0.5 mL) and ethidium homodimer-1 (2.0 mL) were dissolved in 997.5 µL PBS, added to the samples, and incubated for 30 min in the dark at 37 °C in a humidified atmosphere with 5% CO_2_. The samples were photographed under fluorescent live cell microscope with a 10× objective (CTR 6000, Leica, Wetzlar, Germany). Also, the quantitative analysis of LIVE/DEAD assay was measured based on fluorescent intensity percentage (%) using ImageJ (NIH, Bethesda, MD, USA).

### 2.4. Material Characterizations

#### 2.4.1. Scanning Electron Microscopy (SEM)

The fracture site and surface microstructure of 3D-printed composite resin specimens after the flexural strength test was examined using a scanning electron microscope (SEM) (FE-SEM JSM6701F, Jeol Ltd., Tokyo, Japan) at different magnification; 40×, 150×, and 1000×. Before the examination, samples were cleaned using plasma cleaner and then coated with gold–palladium to prevent surface charging and provide a homogeneous surface for analysis and imaging.

#### 2.4.2. Energy-Dispersive X-ray Spectrometer (EDS)

One sample per group was characterised for surface elemental composition via a microscope coupled with an energy-dispersive X-ray spectrometer (FE-SEM JSM6701F, Jeol Ltd., Tokyo, Japan) to ensure the presence of reinforced glass fillers and zirconia glass nanoparticles. The primary electron energy was 20 keV. Two different areas were selected for each sample.

### 2.5. Statistical Analyses

The one-way ANOVA test was employed to assess whether there were any significant differences in flexural strength and cell viability (biocompatibility) among the 3D-printed resin materials under different reinforcement conditions. Levene’s test was used to verify the homogeneity of variance in the data. All statistical analyses were conducted at a significance level of 0.05. Tukey’s honestly significant difference (HSD) test was utilised for pairwise comparisons of means. A 95% confidence interval was calculated.

## 3. Results

### 3.1. Flexural Strength

**Figure 2:**
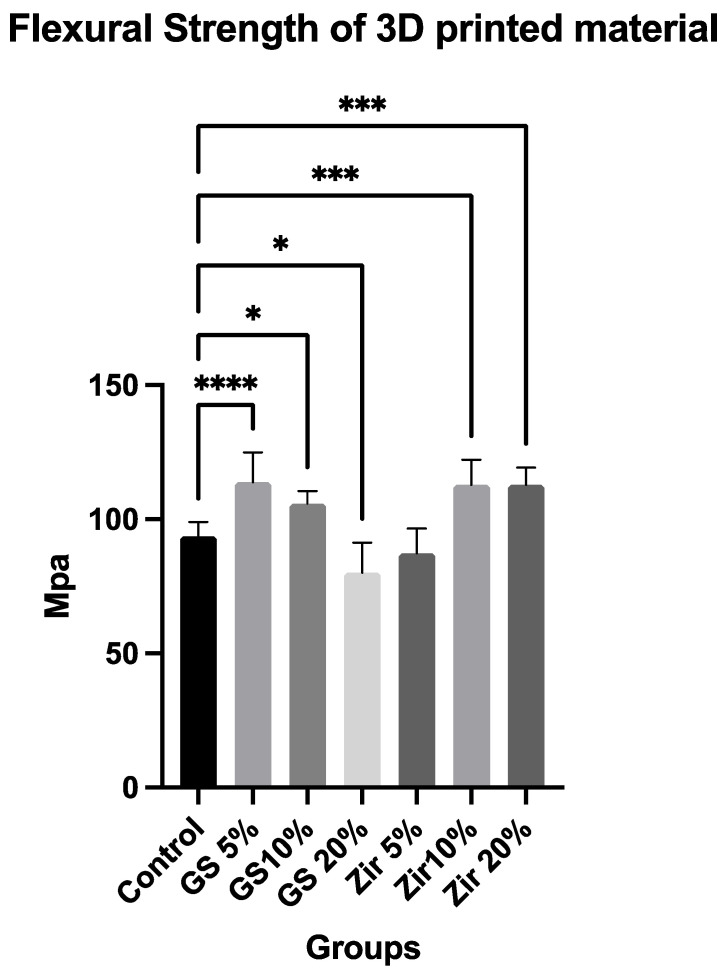
Flexural strength values and comparison between the tested group: control (unmodified 3D-printed resin), 5% glass silica. 10% glass silica, 20% glass silica, 5% zirconia, 10% zirconia, 20% zirconia. Star symbol indicates statistically significant difference between groups (*p* < 0.05).

The results of the flexural strength data are presented in Figure 2, showing the mean values and standard deviation (SD) of the tested groups. A 95% confidence interval was calculated for 10 samples each group. The results of the one-way ANOVA indicate that there are significant differences between the groups (*p* = 0.03). This suggests that the flexural strength of the 3D-printed composite resin material is significantly affected by the glass silica and zirconia reinforcement. This finding is further validated through a Tukey’s HSD post hoc test, which demonstrates that there are statistically significant variations among the groups examined. The mean values of the three-point bending test results range from 80.02 to 113.80 MPa for all tested groups. When compared with the control group (93.69 MPa ± 5.22), significant improvements in flexural strength are observed in groups with 10% zirconia reinforcement (112.80 MPa ± 9.30), 20% zirconia reinforcement (112.90 MPa ± 6.42), 5% glass silica reinforcement (113.80 MPa ± 11.03), and 10% glass silica reinforcement (114.60 MPa ± 4.74). However, there is no significant improvement in flexural strength in the group with 5% zirconia reinforcement (87.36 MPa ± 9.19 Notably, there is a significant decrease in flexural strength in the group with 20% glass silica reinforcement (80.02 MPa ± 11.35). These findings suggest that the addition of zirconia reinforcement in 3D-printed resin composite material significantly improves flexural strength, whereas the effect of glass silica reinforcement is more nuanced and depends on the concentration. These results provide valuable insights for optimizing the mechanical properties of 3D-printed composite resin materials for specific applications.

### 3.2. Cell Viability (MTT Assay)

**Figure 3:**
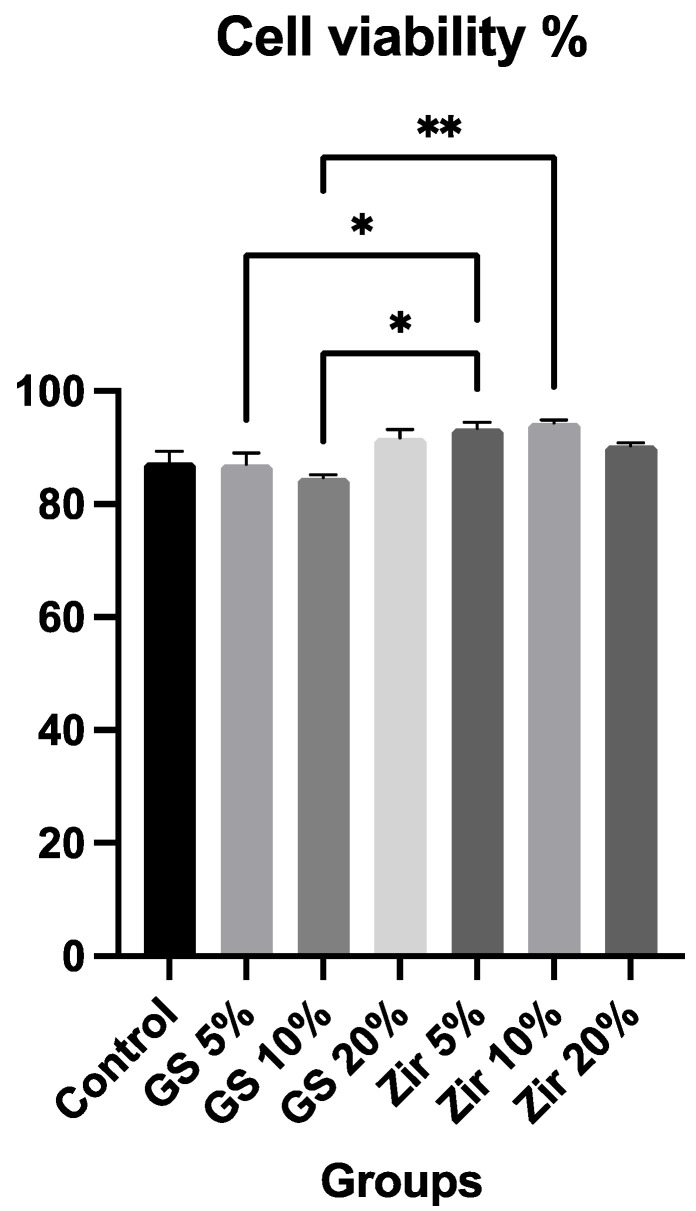
Cell viability (MTT assay) in control and in 3D-printed reinforced resin material groups: control (unmodified 3D-printed resin), glass silica 5 wt% (GS 5%), glass silica 10 wt% (GS 10%), glass silica 20 wt% (GS 20%), zirconia 5 wt% (Zir 5%), zirconia 10 wt% (Zir 10%), and zirconia 20 wt% (Zir 20%). Star symbol indicates statistically significant difference between groups (*p* < 0.05).

The real-time cell viability measurement of human gingival fibroblasts in the presence of printed resin extracts over 24 h is shown in Figure 3. The one-way ANOVA shows statistically significant differences among the groups (*p* = 0.0021), and 95% confidence interval was calculated. The zirconia 10% group has the highest cell viability value (94.33%), whereas the glass silica 10% (84.67%) group exhibits the lowest cell viability value. Furthermore, the results show that the relative viabilities of all groups are greater than 80%, indicating that these printed resins are nontoxic [44]. Residual organic solvent, unreacted monomer leaching, and photo-initiators are all potential sources of cytotoxicity [45]. However, the current study’s MTT assay results indicate that the sources of toxic effects, as mentioned above, are negligible.

### 3.3. LIVE/DEAD Staining for Live-Cell Imaging

**Figure 4:**
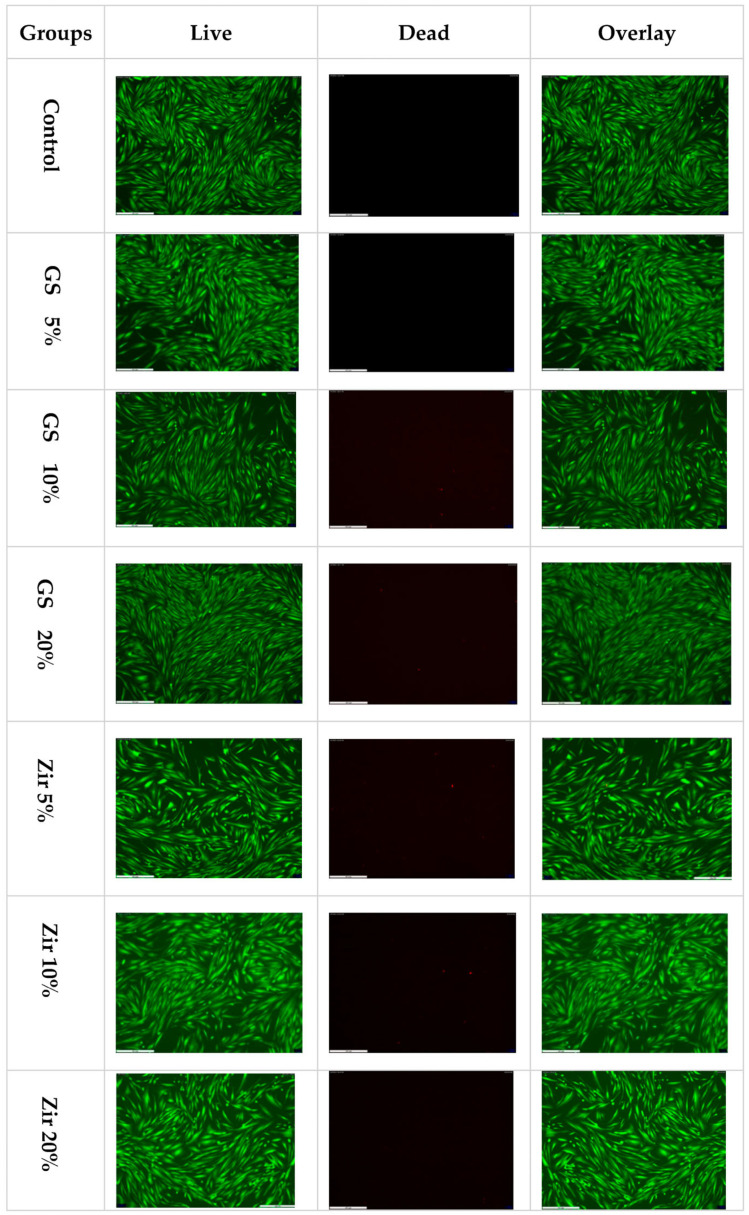
LIVE/DEAD staining of 3D-printed temporary crown material treated after indirect contact with gingival fibroblasts. Images represent live/dead assay of all groups tested as follows: control (unmodified 3D-printed resin), glass silica 5 wt% (GS 5%), glass silica 10 wt% (GS 10%), glass silica 20 wt% (GS 20%), zirconia 5 wt% (Zir 5%), zirconia 10 wt% (Zir 10%), and zirconia 20 wt% (Zir 20%).

**Figure 5:**
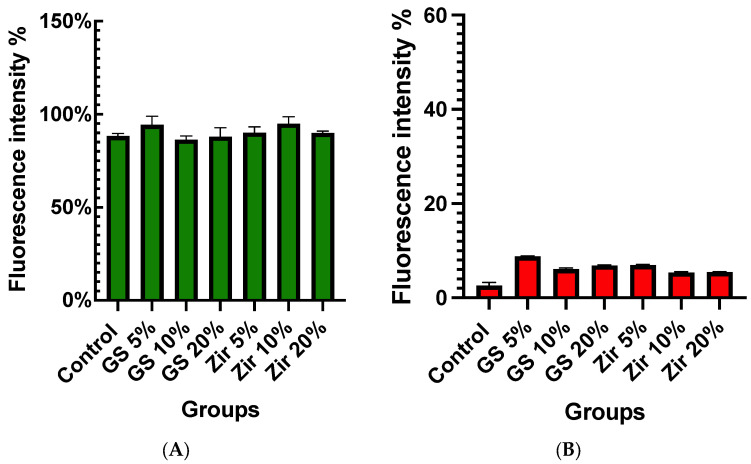
Bar graph showing the total fluorescent intensity (%) of live/dead cells of all groups tested: (**A**) the total fluorescent intensity (%) of live cells; (**B**) the total fluorescent intensity (%) of dead cells.

Staining cells with calcein AM and ethidium homodimer-1 (Molecular Probes, Eugene, OR, USA) and observing the MTT assay results with fluorescent microscope provides additional confirmation (CTR 6000, Leica, Germany). Live cells fluoresce intensely green, whereas dead cells fluoresce intensely red (Figure 4). The fluorescent intensity percentages were used to measure the quantitative results of the LIVE/DEAD assay (Figure 5). The values range between 86.44% to 94.92%. Among the groups, the zirconia 10% group shows the highest mean fluorescent intensity of live cells (94.92% ± 1.41), while the glass silica 10% group exhibits the lowest mean value (86.44% ± 1.92). As for the fluorescent intensity of dead cells, the percentage ranges from 2.56% for the control group to 7.43% for the glass silica 10% group. The Live/Dead Assay^®^ image assay results match those of the MTT assay and results test.

### 3.4. SEM Analyses

**Figure 6:**
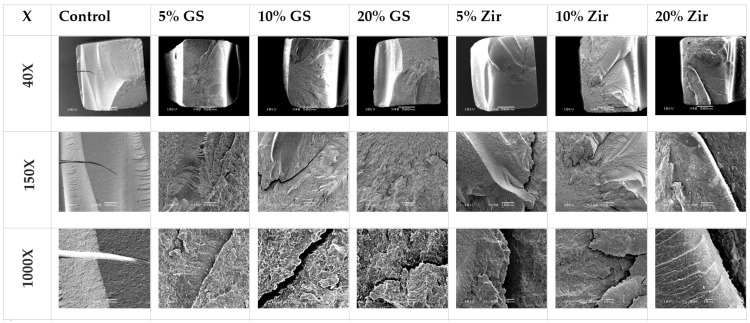
Representative SEM images (magnification 40×, 150×, and 1000×) of the fracture surface of tested groups: control, glass silica 5 wt% (GS 5%), glass silica 10 wt% (GS 10%), glass silica 20 wt% (GS 20%), zirconia 5 wt% (Zir 5%), zirconia 10 wt% (Zir 10%), and zirconia 20 wt% (Zir 20%).

All 3D-printed resin samples exhibit similar fracture patterns after three-point bending strength testing. The SEM microstructural investigations of the fractured surfaces of the control and reinforced specimens are shown in Figure 6 at a magnification of 40, 150, and 1000×. Overall, 3D-printed resin that is reinforced with nanoparticles exhibits slightly rougher fracture surfaces around the crack site than the control (unmodified) sample. The 3D-printed resin with 5%, 10%, and 20% glass fillers has a ductile fracture mode when compared with the samples that are reinforced with zirconia glass at all concentrations (5%, 10%, and 20%), which has a more brittle fracture. The control groups display a nearly smooth, broken surface with little resistance, where the fractured surfaces are typically well-defined, flat, compact, and organised. The incorporation of glass silica (GS) and zirconia glass (ZG) at varying concentrations leads to significantly more irregular surface patterning. The surface topography has a flake-like appearance, lamellae, and fissure-like structures. The fracture pattern results in a more elevated and rougher surface at the crack site, indicating an increase in ductility. An unfavourable fracture could have taken place in the specimens reinforced with 5%, 10%, and 20% GS, as evidenced by the presence of voids and cracks on the fractured surface. This may have been caused by the aggregation of the nanofiller, which acts as a defect in the polymer matrix structure. This may lead to a poor level of interaction between the GS and ZG nanoparticles and the 3D-printed resin matrix.

### 3.5. EDS Analyses

**Figure 7:**
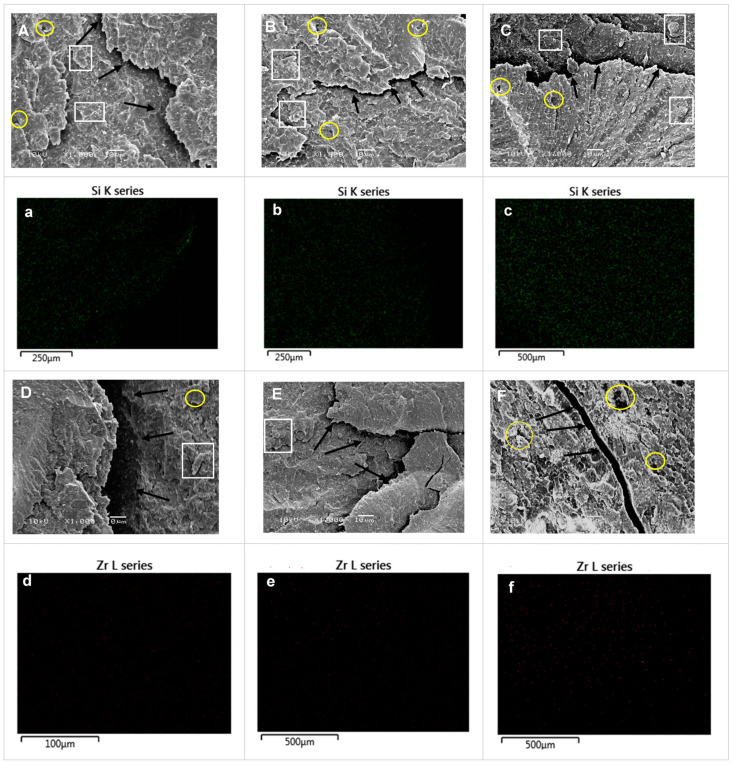
SEM images (high magnification ×1000) of the fractured surfaces of 3D-printed resin that shows the distribution of glass fillers and zirconia glass nanoparticles after embedding with 3D-printed resin with different concentrations: (**A**) 5% glass silica; (**B**) 10% glass silica, (**C**) 20% glass silica; (**D**) 5% zirconia, (**E**) 10% zirconia, (**F**) 20% zirconia. Element mapping images of glass fillers (**a**–**c**) and zirconia glass (**d**–**f**) element in the 3D-printed resins. Notable features are indicated for (**A**–**F**): microcracks (marked by black arrows), voids (highlighted with yellow circles), and clusters (identified by white rectangular). These observed features offer valuable insights into the structural characteristics and failure mechanisms of the reinforced 3D-printed resin.

**Figure 8:**
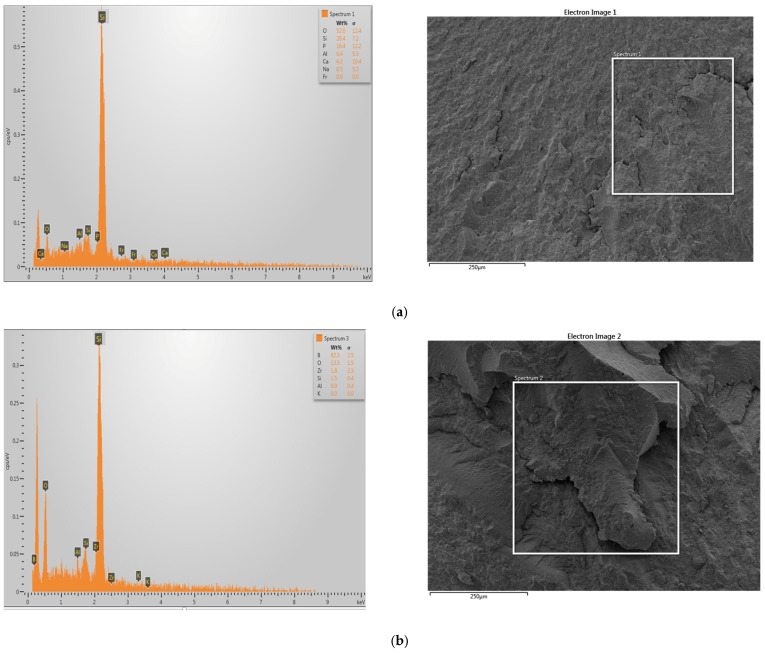
SEM image, EDS spectrum, and element distribution of nanoparticles that used to modify the 3D-printed resin. (**a**) Glass-silica-reinforced 3D-printed resin, (**b**) zirconia-glass-reinforced 3D-printed resin.

The composition of 3D-printed resin reinforced with glass fillers or zirconia glass was analysed using an energy-dispersive X-ray spectrometer (EDX). The SEM images and EDX element mapping images in Figure 7 demonstrate the presence of zirconia and glass filler elements in the samples modified with GS or ZG. The distribution of modified glass filler and zirconia nanoparticles within 3D-printed resin was observed using an SEM–EDS mapping technique, as shown in Figure 7a–c for the glass fillers and Figure 7d–f for the zirconia glass. The red-coloured marks in the image indicate the presence of ZrO_2_ nanoparticles on the surface, as detected by the EDS mapping technique. The green spots represent the presence of silica elements on the sample surface. As the density of added nanoparticles increases, the number of spots on the surface also increases (Figure 7). The EDS spectra and the corresponding elemental compositions, shown in atomic and weight percentages in Figure 8, confirm the presence of silica in the sample reinforced with glass filler and the presence of zirconia in the sample reinforced with zirconia glass.

## 4. Discussion

The incorporation of additive technology in dentistry, such as 3D-printing, has generated significant interest among researchers. Evaluating the mechanical properties and biocompatibility of 3D-printed materials compared to conventional counterparts is essential to determine their suitability for long-term clinical use in dental restorations. A thorough investigation of these factors can contribute to advancing the field of dental materials and optimizing patient care in dentistry.

In recent times, dental research has concentrated on enhancing the quality of 3D-printed dental materials, particularly dental bridges and crowns, so that they can be effectively utilised in clinical practice. This entails improving their biocompatibility and durability [46,47]. The material’s mechanical properties and biological compatibility are crucial, as they can impact the dental prosthesis’s long-term performance [48]. In addition, the degree of polymerisation, addition of reinforcing materials, and the printing parameters can also affect the quality of the final product [49,50,51]. Therefore, careful evaluation of these factors is crucial for selecting suitable dental materials. Mechanical properties, such as flexural strength, are essential to withstand chewing forces. This is particularly important for 3D-printed temporary restorations used for extended periods before the final restoration is fabricated [14,52].

One technique that has proven effective in enhancing the flexural strength and other attributes of dental resin composites is the inclusion of nanoparticle fillers [14,53]. This approach has also been shown to enhance tensile strength and wear resistance [54], and elastic modulus, as well as a reduction in the polymerisation shrinkage of the material, among other properties [55]. This in vitro study evaluated the effect of the reinforced PMMA–glass fillers and zirconia composite 3D-printed specimens of various weight ratios on this material’s flexural strength and biocompatibility properties. The first null hypothesis that the addition of nanoparticles in the 3D-printed resin would have no statistically significant difference in mean values compared to the control 3D-printed resin was rejected. The second null hypothesis was also rejected based on the results of the MTT assay and the observation of statistically significant differences in fibroblast cell viability.

It is interesting to note that 3D-printed temporary materials may have lower flexural strength than other materials and that efforts to improve the mechanical properties of 3D-printed materials may be necessary to make them suitable for use as permanent crowns. It is common for 3D-printed materials to have lower flexural strength than other materials, such as materials produced by computer-aided design/computer-aided manufacturing (CAD/CAM) and PMMA resin. This is because a material’s strength depends on various factors, including its composition, manufacturing process, and microstructure. For example, 3D printing involves building up an object layer-by-layer using various materials, including plastics, metals, and ceramics. The way the materials are layered by these layers can result in a microstructure that is less homogeneous and more porous than materials produced using other manufacturing methods, such as casting or machining. This can lead to lower strength and other mechanical properties.

Based on the present study’s findings, the flexural strength of 3D-printed resins increases with the addition of nanoparticles, and this increase is shown when zirconia nanoparticles are added at concentrations of 10% and 20%, and when glass fillers are added at concentrations of 5% and 10%. This agrees with previous studies investigating the effect of different nanoparticles on 3D-printed resins [14,22]. The flexural strength results ranges from 80.02 to 113.80 MPa for all tested groups, and these results are comparable with other studies on temporary resin-based materials. However, the findings from the flexural strength test indicate that the 3D-printed temporary material has a lower flexural strength in comparison to the CAD/CAM and conventional PMMA resin materials studied in earlier research [42,50,56]. The mean values of their studies for additive manufacturing, CAD/CAM, and conventional materials are 79.54 MPa, 104.20 MPa, and 95.58 MPa, respectively. These values are comparable to the flexural strengths of our reinforced 3D-printed resin, which range from 80.02 to 113.80 MPa in other studies [57,58].

The addition of zirconia and glass filler nanoparticles to a resin matrix for use in 3D-printed denture-base materials appears to increase the strength of the material. This may be due to the homogenous distribution of the nanoparticles within the resin and their ability to make the materials more resistant to cracking when subjected to load. This enhancement is explained by the presence of reinforcing fibres within a resin–composite material, increasing overall strength by changing stress dynamics and increasing filler size and fraction [59]. Using glass silica and zirconia nanoparticles to reinforce materials can reduce crack propagation and improve stress distribution when tested in a three-point bending configuration [60]. These improvements can be attributed to several factors, including the small diameter of the nanoparticles and their ability to distribute stresses more effectively within the material [61]. The current study finds that the flexural strength of 3D-printed resins modified with zirconia glass and glass filler nanoparticles exceeds the ISO recommendations of 65 MPa. Furthermore, the study finds that certain modified groups of glass filler (5% and 10%) and zirconia glass (10% and 20%) have even higher values than the control (unmodified) 3D-printed resin. This is a positive result that justifies additional research to explore the long-term clinical effects of this modification. The lack of significant improvement in flexural strength with 10 wt% and 20 wt% of glass fillers, despite the observed improvement with zirconia particles, could be attributed to factors such as particle distribution and filler content–mechanical property relationship [62]. Studies conducted in the past demonstrate that changes in the ratio of nanoparticles/fibres can positively or negatively impact dental resins’ surface hardness and flexural strength [63,64]. It is possible that the distribution of the glass fillers is less homogeneous at higher concentrations (10 wt% and 20 wt%) compared to 5 wt%, resulting in uneven dispersion or agglomeration of fillers [65]. This non-uniform distribution of fillers can create stress concentration points and weaken the material, leading to reduced flexural strength [66,67]. Another possible explanation is that the relationship between filler content and mechanical properties of composite materials is often not linear. There could be an optimal filler content range for achieving the best mechanical properties and exceeding that range may result in diminished effects or even adverse effects on the properties, which has been mentioned in previous studies [68,69]. It is possible that 5 wt% of glass fillers fall within the optimal range, while 10 wt% and 20 wt% exceed it, leading to reduced or negligible improvements in flexural strength. Further investigations and optimization of these factors, including particle distribution, and the relationship between filler content and mechanical properties, may be necessary to better understand and explain the observed findings in a more comprehensive manner within the context of academic research.

Figure 5 presents the SEM images of groups reinforced with glass filler (5% and 10%) and zirconia glass (10% and 20%). As can be observed, these materials display brittle fractures with visible cracks on their surface. These cracks are the result of force being applied. When small, durable crystalline particles, such as zirconia or glass filler nanoparticles, are evenly dispersed within the resin matrix, it becomes more difficult for the crack to pass through these particles as opposed to passing through the resin matrix alone. This is referred to as “dispersion strengthening”, which effectively hinders the spread of cracks through the resin structure [70,71].

The groups reinforced with 20% weight glass silica nanofiller have a surface that is characterised by the presence of multiple voids and cracks. These defects are likely caused by the agglomeration of the nanofiller within the polymer matrix structure [72]. The agglomeration of the nanofiller acts as a weak point within the matrix, making the material more susceptible to fracture. Furthermore, the high percentage of nanofiller used in the material likely results in poor adhesion between the untreated nanofiller and the polymer matrix. This poor adhesion also contributes to the undesirable fracture of the material.

In the current study, the biocompatibility of 3D-printed reinforced dental resin was investigated by analysing the viability of fibroblast cells. Most previous studies on biocompatibility conducted bacteria-related in vitro cell experiments on temporary restorative material [73,74]. However, few in vitro tests used various cell types from tooth-related tissues, such as oral keratinocytes, gingival fibroblasts, and periodontal ligament cells, to examine the biological effects of resin materials on human tissues [75,76,77]. The current findings do not show any significant effects on cell proliferation, emphasizing the importance of adhering to the manufacturer’s guidelines when purchasing materials intended for intraoral use, reducing the possibility of side effects caused by, for example, incomplete resin polymerisation and the presence of residual monomers.

Figure 4 indicates that the nanoparticle fillers added to the 3D-printed resin significantly impact cell viability. Cell viability values range from 87% to 94% compared to the positive control (100%). Additionally, adding ZrO_2_ nanoparticles does not affect cell proliferation and shows significantly higher viability for all groups (5%, 10%, and 20%). Zirconia groups also show higher cell viability than glass silica groups. This may be connected to the more uniform distribution of filler, which can decrease the proportion of the organic phase and, subsequently, decrease the amount of unpolymerised monomer [78,79]. Our findings are consistent with previous research, which found that modifying 3D resin with nanoparticles can result in the development of a material with a better biocompatibility property [14,80,81]. This suggests that combining zirconia and glass silica nanoparticles with 3D-printed provisional dental restoration materials could create effective nanoparticles that would lead to biocompatible reinforced dental material. However, more research is needed to fully understand the potential advantages and disadvantages of using nanoparticles in this way, and to confirm the safety and effectiveness of these materials for dental restoration.

The findings of this study indicate that nanoparticle-enhanced 3D-printed crown resin could be beneficial in a clinical setting. This type of resin may overcome some of the limitations of traditional 3D-printed resin, such as its mechanical properties or biocompatibility. Additionally, using nanoparticles in the resin helps improve the material’s clinical applicability, making it more suitable for various dental applications. However, more research is needed to fully understand the potential benefits of nanoparticle-enhanced 3D-printed resin in clinical settings. This study is not without its limitations. First, the geometry of the samples used in this work differs from that used in clinical settings. Therefore, crown-shaped samples must be fabricated to simulate the clinical scenario. Another limitation is using two different-sized nanoparticles for dental resin reinforcement. This choice complicates evaluating and comparing their individual effects on resin properties. Using nanoparticles of the same size would allow for a more direct comparison. However, the rationale behind selecting different-sized nanoparticles was to explore the potential influence of nanoparticle size variation on the reinforcement capabilities of the dental resin. Several factors can affect the mechanical properties of 3D materials printed using digital light processing (DLP) technology. These factors include the printing orientation, water absorption level, long-term survival rate, colour stability, and fatigue behaviour of the 3D-printed material. Having a deep understanding of these factors is indispensable for creating crown and bridge materials that are suitable for extended clinical use, as well as for improving the dependability and predictability of dental treatment procedures that incorporate 3D-printed crown and bridge materials. This knowledge can enable dental practitioners to make informed decisions, thereby enhancing the quality of patient care [38,46].

## 5. Conclusions

The results of this in vitro study can conclude the following:

1. The addition of ZrO_2_ nanoparticles and 5 and 10 wt% glass fillers shows increased flexural strength of 3D-printed resin;
2. Incorporating zirconia and glass fillers nanoparticles creates a biocompatible 3D-printed resin that does not negatively affect cell viability.

## Figures and Tables

**Table 1:** Material compositions that used in this study.

Material	Compositions (Wight%)	Manufacturer
Everes Temporary (dental resin)	Aliphaticdifunctional methacrylate < 50% 2.2 ethylenedioxydiethyl dimethacrylate < 40% Aliphatic unethane acrylate < 20% Phosphine oxide < 2.5% Ethylenedioxydiethyl dimethacrylate < 40% Aliphatic unethane acrylate < 20%	Sisma, Italy
Glass fillers (ultrafine GM35429)	SiO_2_ < 30%, CaO < 10%, Al_2_O_3_ < 30%, F < 15%, P_2_O < 10%, Na_2_O < 10%	Shofu Inc., Ratingen, Germany
Zirconia glass (ultrafine GM018-307)	Al_2_O_3_ < 5.0%, B_2_O_3_ < 15%, K_2_O < 5%, SiO_2_< 65%, ZrO_2_ < 5.0%	Shofu Inc., Ratingen, Germany

## Data Availability

The data presented in this study are available on request from the corresponding author.
